# The effect of the COVID-19 pandemic on deceased and living organ donors in the United States of America

**DOI:** 10.1038/s41598-022-24351-x

**Published:** 2022-11-30

**Authors:** Mireille Hantouche, Libia Lara Carrion, Emilio Porcu, Katrina A. Bramstedt

**Affiliations:** 1 Department of Mathematics, Khalifa University, Abu Dhabi, United Arab Emirates; 2 School of Medicine, Khalifa University, Abu Dhabi, United Arab Emirates; 3 Bond University Medical Program, Robina, QLD Australia

**Keywords:** Epidemiology, Diseases, Health care, Mathematics and computing, Scientific data, Statistics

## Abstract

A life-saving treatment, solid organ transplantation (SOT) has transformed the survival and quality of life of patients with end-organ dysfunction. The coronavirus disease (COVID-19) pandemic has impacted the practice of deceased and living donations worldwide by various resource shifting, including healthcare personnel and equipment such as ventilators and bed space. Our work explores the COVID-19 pandemic and global transplant data to create a statistical model for deducing the impact of COVID-19 on living donor and deceased donor transplants in the United States of America (USA). In severely impacted regions, transplant centers need to carefully balance the risks and benefits of performing a transplant during the COVID-19 pandemic. In our statistical model, the COVID cases are used as an explanatory variable (input) to living or deceased donor transplants (output). The model is shown to be statistically accurate for both estimation of the correlation structure, and prediction of future donors. The provided predictions are to be taken as probabilistic assertions, so that for each instant where the prediction is calculated, a statistical measure of accuracy (confidence interval) is provided. The method is tested on both low and high frequency data, that notoriously exhibit a different behavior.

## Introduction

Organ transplant is an indispensable surgical technique for improving patient quality of life, as well as survival^1^. A prior study analyzed 25 years of transplant data and found that 2,270,859 life years were saved in 533,329 patients through 2012^2^. According to Global Observatory on Donation and Transplantation (GODT), actual deceased organ donors per million population (pmp) in 2019 were as follows: Donors after Brain Death (DBD) 31,366 and Donors after Cardiac Death (DCD) 9242. Globally, this equates to 153,863 solid organ transplants (Kidney 100,097, Liver 35784, Heart 8722, Lung 6800, Pancreas 2323, and S. bowel 137)^3^. Donation rates vary by country due to various reasons including religious and cultural perspectives, health system design (e.g., types of donation, inclusion of family refusal of donor consent), education and promotion efforts, etc. Spain is known to have the highest rates of organ donation in the world due to a combination of factors such as community education, as well as streamlined logistical operations (i.e., staff, resources, policies, procedures) for donation and transplant^4,5^.

Evidence shows that the need for transplantation is constantly increasing (Matesanz et al.^6^; GODT (WHO)^3^), and donor organs are consistently in short supply. Recently, GODT data indicates worldwide, only 10% of waiting lists are transplanted^7^. Adding to this strain is the COVID-19 pandemic caused by the severe acute respiratory syndrome coronavirus type 2 (SARS-CoV-2) virus. Specifically, on 30 January 2020, the WHO Emergency Committee declared a global health emergency^8^. On November 24, 2021, a new rapidly spreading variant of SARS-CoV-2, B.1.1.529 (Omnicom), was reported to the World Health Organization (WHO). COVID-19 impacts patients’ organ systems and often results in organ failure, thereby increasing transplant waiting lists.

Donation from asymptomatic individuals who have been in a COVID-19-affected area in the last 28 days is discouraged^9^. Asymptomatic individuals being monitored following contact with a proven case of COVID-19 are excluded from donation^9^. Finally, individuals being tested for COVID-19 are excluded unless they are SARS-CoV-2 negative; however, recent research indicates that COVID-19 positive donors have been used for kidney transplants with favorable outcomes^9^ (these donors were known to be positive at the time of donation).

Between January 1 and March 12, 2020 in the USA, the mean number of deceased organ donors was 7.2/day, and the mean number of organ transplants was 16.1/day (15.2 transplants from deceased donors per day)^10^. During the first 6 weeks after the WHO emergency was declared, deceased donations dropped to 1.2/day and transplants dropped to 2.1/day^10^.

Our work explores the COVID-19 pandemic and global transplant data to create a statistical model for deducing the impact of COVID-19 on living donor and deceased donor transplants in the USA. This model acknowledges the complex setting of the COVID-19 pandemic whereby staffing shifts have occurred, moving clinical personnel away from transplant to other areas such as emergency medicine and intensive care medicine. Also, the need for critical care beds and ventilators can take resources from transplants, shifting them to COVID-19 care. In severely impacted regions, transplant centers need to carefully balance the risks and benefits in performing a transplant during the COVID-19 pandemic^11^.

In 2021, Suarez-Pierre et al.^12^ measured the variation between observed and expected rates of solid organ transplantation during the COVID-19 pandemic using an autoregressive integrated moving average (ARIMA) model focused on monthly time points. In the current study, the ARIMA model is also used to explore the impact of COVID-19 on deceased and living donation; however, the weekly time points (rather than monthly) allow for increased sensitivity to identifying volume shifts, and thus potentially improved forecasting.

## Methodology

Using statistical techniques, we studied the time series of deceased and living organ donors in the United States (US) during the COVID-19 pandemic (1988 until 2021).

### Data sources

Data were obtained from the United Network for Organ Sharing/UNOS database and Organ Procurement and Transplantation Network/OPTN metrics^13^. OPTN datasets (https://optn.transplant.hrsa.gov/data/about-data/) are anonymized and the researchers have no links to patients’ personal identifying information. All methods were conducted in accordance with pertinent guidelines and regulations. We used this open resource de-identified national dataset, therefore no Institutional Review Board (IRB) or informed consent was required.

### Data variables, sample size, and sampling type

From the online OPTN database^13^ two different sets of data were extracted, for both the deceased and living donors (four different time series):

(i) *Set 1* refers to data recorded and reported on an annual basis, from 1988 until 2021.
(ii) *Set 2* refers to data reported on a weekly basis: from week 1 of 2019, until week 3 of January 2022.

### Study design and setting

Descriptive statistics are a first approximation to a basic understanding of raw data (for patterns and trends) under the principle of dimension reduction. That is, the raw data are first resumed with a bunch of indicators to form a preliminary idea about the behaviour of the phenomenon under study.

The descriptive statistics were then sharpened by inspecting the autocorrelation of the four time series at hand. Autocorrelation is a linear measure of concordance or discordance as a function of the temporal separation between pairs of observations. A direct way to visualize autocorrelations is by plotting the empirical autocorrelation values against the temporal lag. These empirical autocorrelation plots can provide valuable insights into the temporal dependence of the observed data. The autocorrelation plots are obtained by the function *acf*^14^ which uses the overall series sample mean and sample variance to determine the correlation coefficient.

### Statistical analysis

The statistical model *ARIMA* (autoregressive integrated moving average) was used to study the behaviour of the time series and to predict future organ donors’ trends in relation to the pandemic. The acronym ARIMA stands for a parametric family of time series models (*p*, *d*, *q*) in which *p* stands for the order in the autoregressive term (termed lag order throughout), *d* for the order of differentiation of the time series, and *q* for the number of terms in the moving average representation. More sophisticated versions of this model are currently available^15^. Amongst many, the formulation ARIMA(*p*, *d*, *q*)(*P*, *D*, *Q*), where the triplet (*P*, *D*, *Q*) is the seasonality counterpart of the triplet (*p*, *d*, *q*) as previously discussed, was used. The Hyndman-Khandakar algorithm^16^ was utilized to optimize the choice of these model parameters, where optimality is loosely understood as a minimization of some loss function. We shall avoid mathematical obfuscation here, and for technical details, the reader is referred to^16^.

## Results

Time series is a collection of data points that are usually equally spaced over a given interval of time. While time series analysis aims at understanding the structure of an underlying, unobserved, stochastic process, time series forecasting^17^ is instead based on using a given mathematical model to predict future values on the basis of a set of available observations from the past. Presented below are the results for the descriptive statistics of the four time series, their corresponding autocorrelation plots, and finally the ARIMA models developed for predictions.

### Descriptive statistics: deceased and living donors data

**Figure 1:**
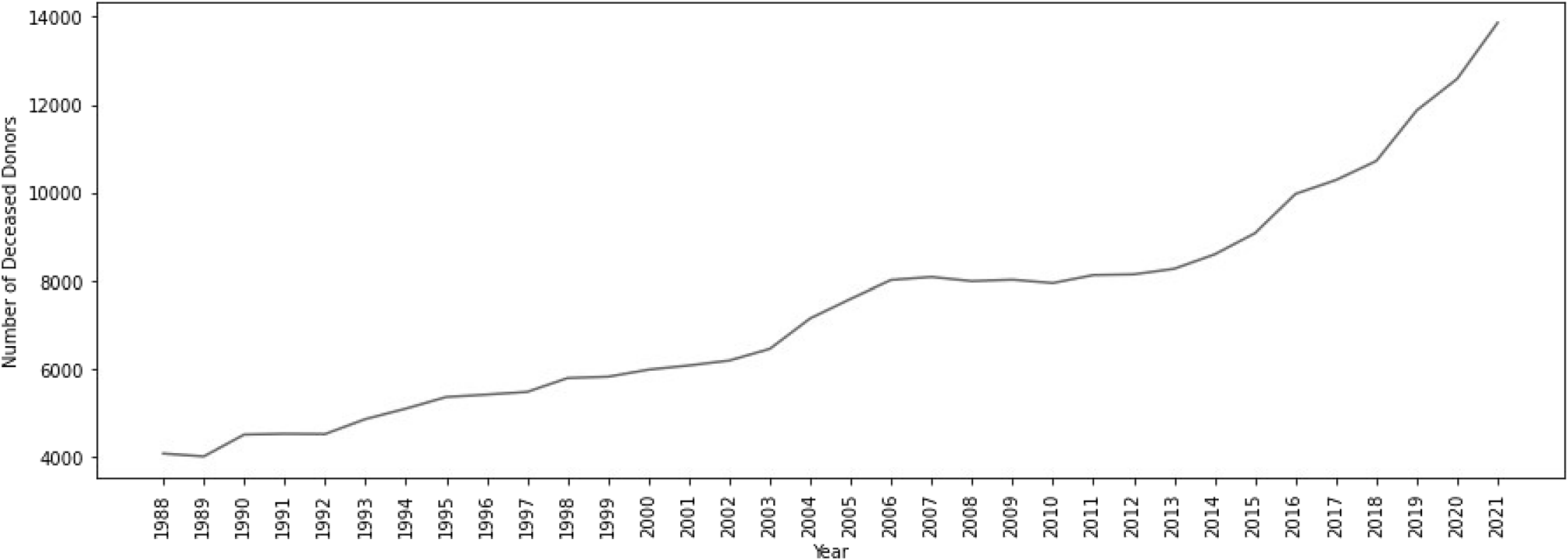
Total number of USA deceased organ donors per year, 1988–2021.

**Figure 2:**
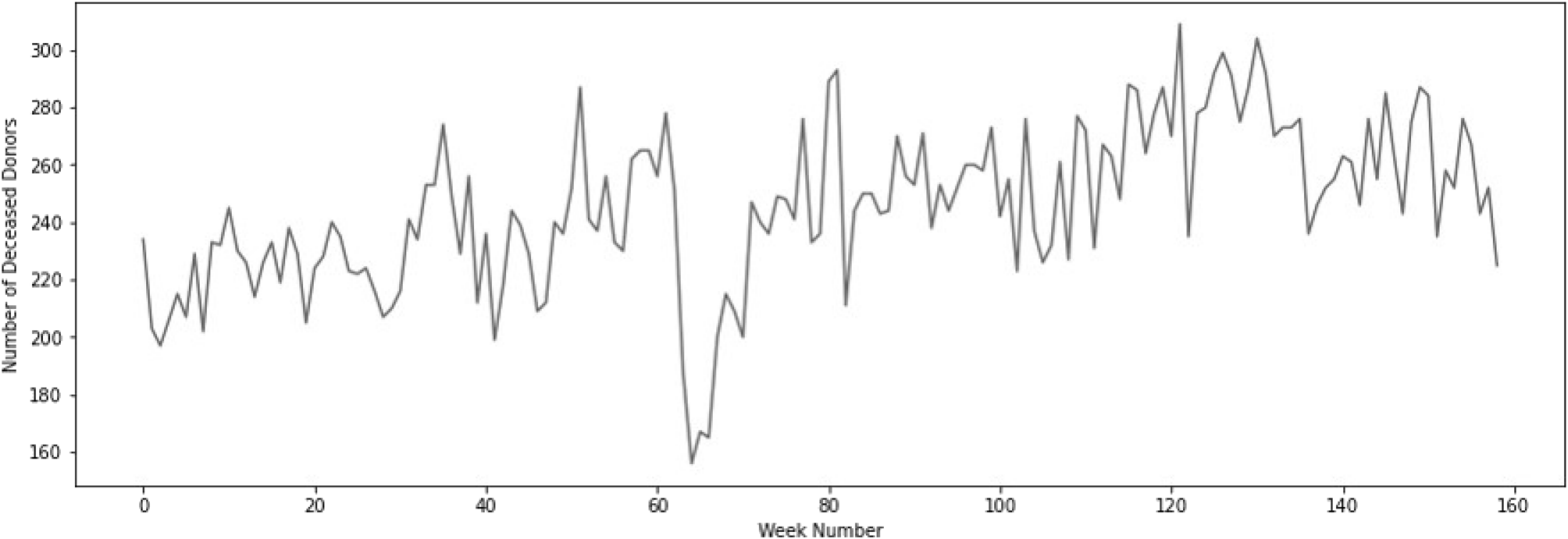
Number of USA deceased organ donors weekly, beginning of 2019 until week 3 in 2022.

**Figure 3:**
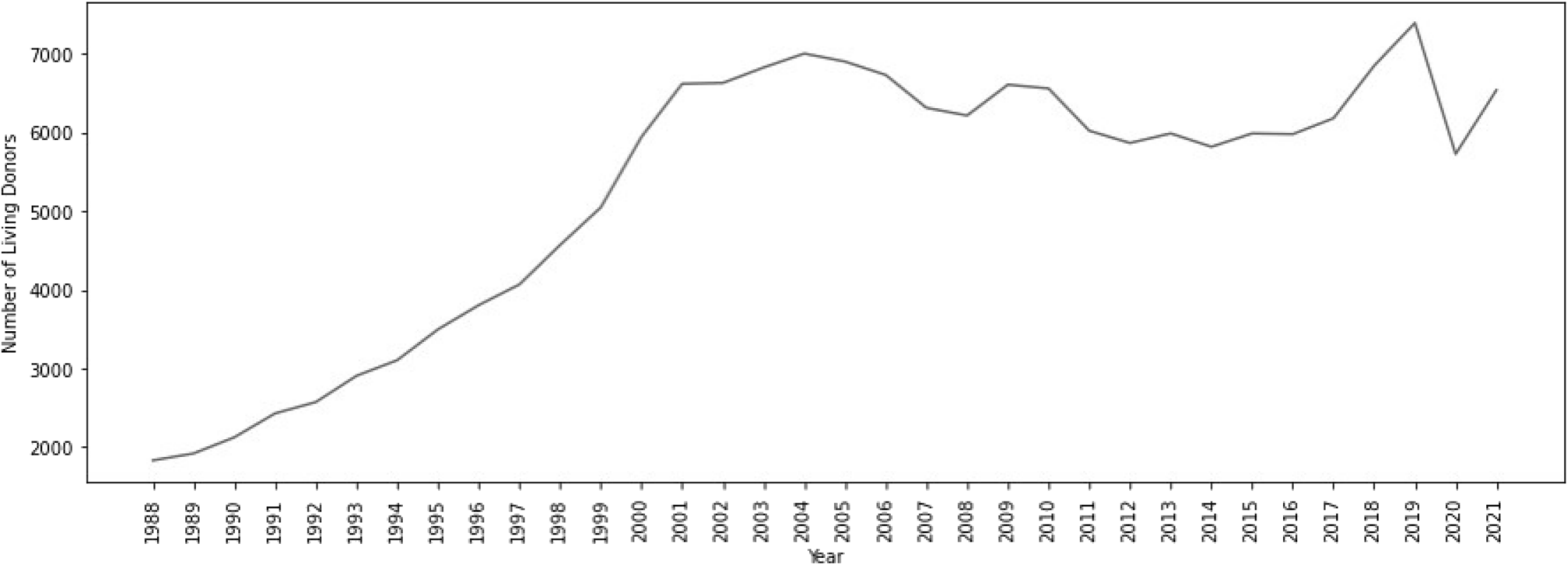
Total number of USA living organ donors per year, 1988–2021.

**Figure 4:**
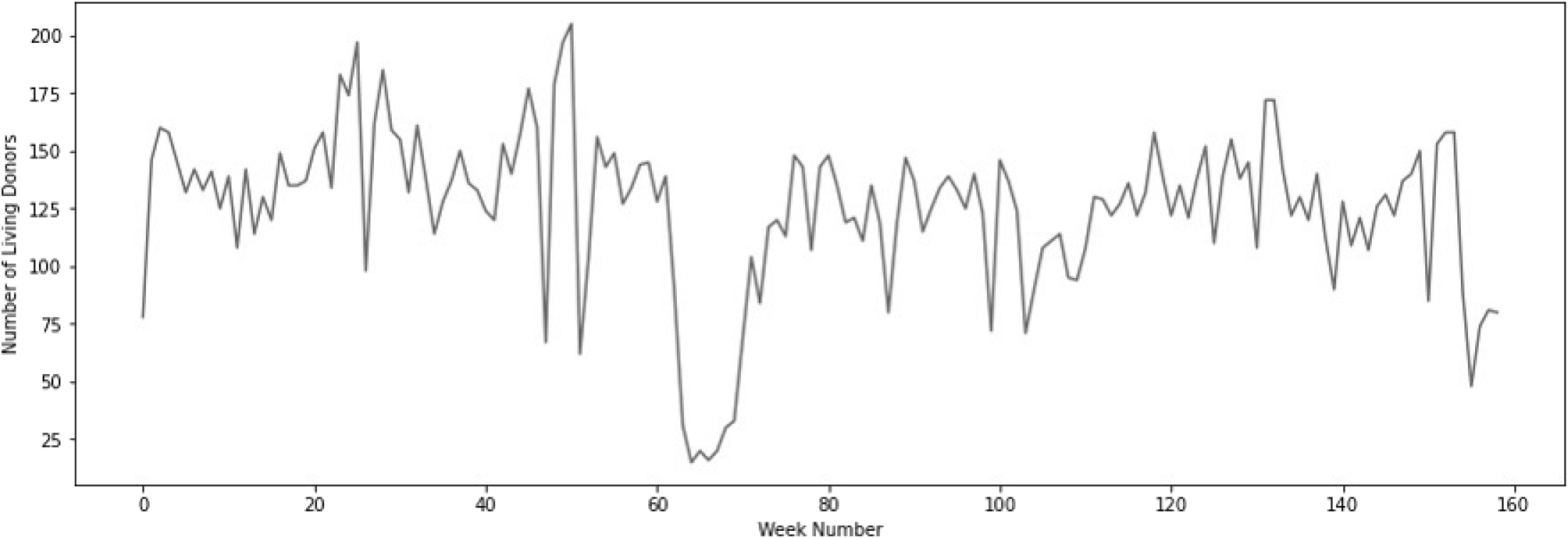
Number of USA living organ donors weekly, beginning of 2019 until week 3 in 2022.

Descriptive statistics for the data of the deceased organ donors and living organ donors are presented in Figs. 1, 2, 3 and 4.

As can be seen in Fig. 1, the number of deceased donors follows an increasing trend, that roughly reaches a sill between 2006 and 2013, but then increases in 2014 and continues to increase even in 2020, when the COVID-19 pandemic began. Referring to the yearly data, the number of deceased organ donors does not appear to be negatively impacted.

Figure 2 presents weekly data, whereby the time span of the COVID-19 pandemic is more clearly visible (as compared to yearly data). A more oscillatory nature of the weekly data is observed when compared to the smooth yearly data. Also, minimum values are observed for weeks 65–67 (or weeks 13–15 in 2020 spanning from March 23 till April 12, 2020).

Yearly data in Fig. 3 indicate that the COVID-19 pandemic had significant impact on USA living organ donation. As shown, donations begin to increase in 2019; however, they decrease considerably in 2020, with a slight increase in 2021.

Weekly data in Fig. 4 detail the decrease in living donations starting from cumulative week number 64 till 71 (or weeks 12–19 in 2020 spanning from March 16 till May 10, 2020).

### Autocorrelation plots

**Figure 5:**
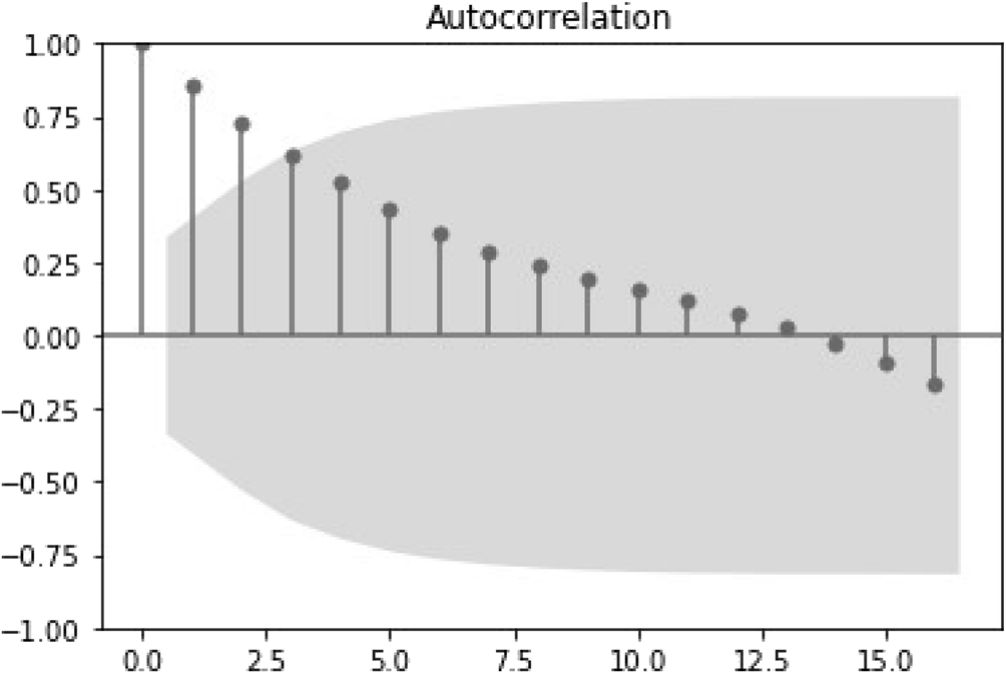
Autocorrelation plot for the time series of the annual deceased donors.

**Figure 6:**
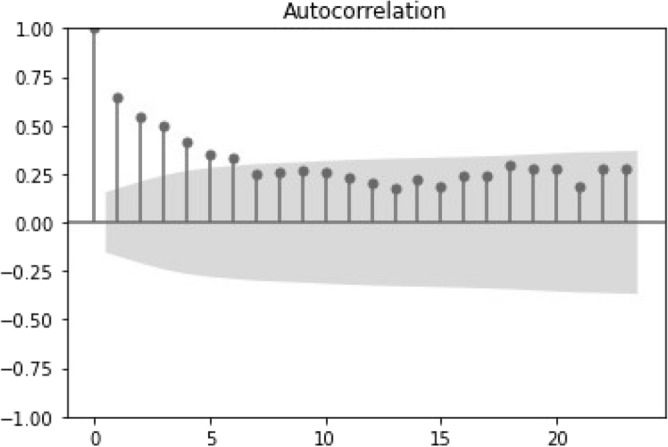
Autocorrelation plot for the time series of the weekly deceased donors.

Autocorrelation is a linear measure of dependence between observations that are separated by given temporal lags. Intuitively, observations that are temporally far apart are less likely to be dependent than observations that are closer. This fact is well known in geographical sciences under the name *Tobler’s law*^18^. Figures 5 and 6 depict the autocorrelation against temporal lags for the number of deceased donors, per year, and per week, respectively. For the yearly time series, the temporal correlation is persistent, as, after many months of temporal separation, the correlation continues strictly positive. Note that after lag 12 the correlation attains negative values, meaning that observations that are more than 15 years far apart tend to be negatively correlated. However, this negative correlation is quite weak.

**Figure 7:**
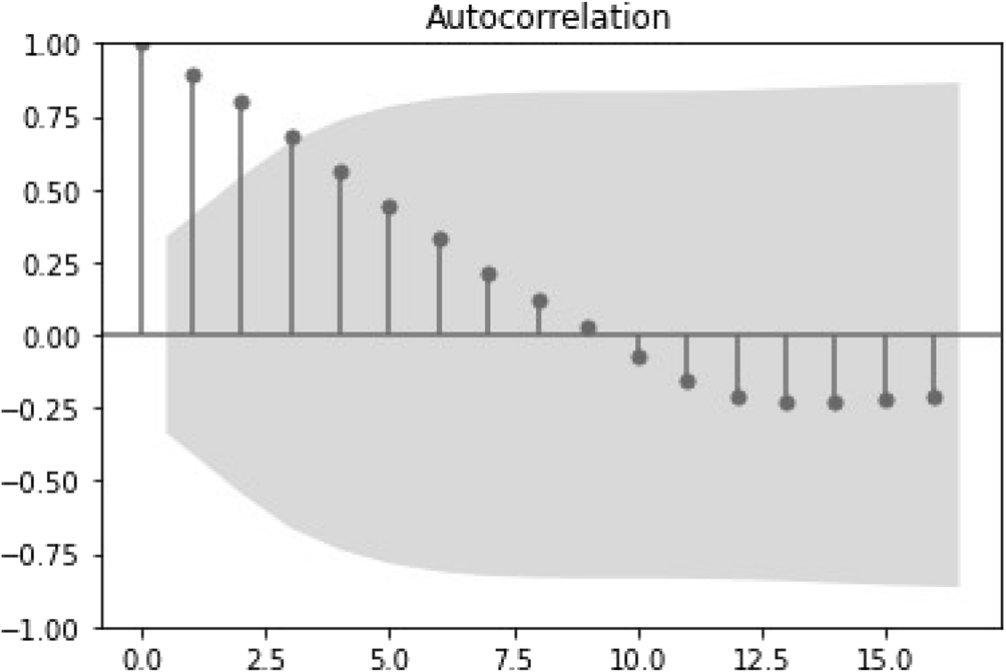
Autocorrelation plot for the time series of the annual living donors.

**Figure 8:**
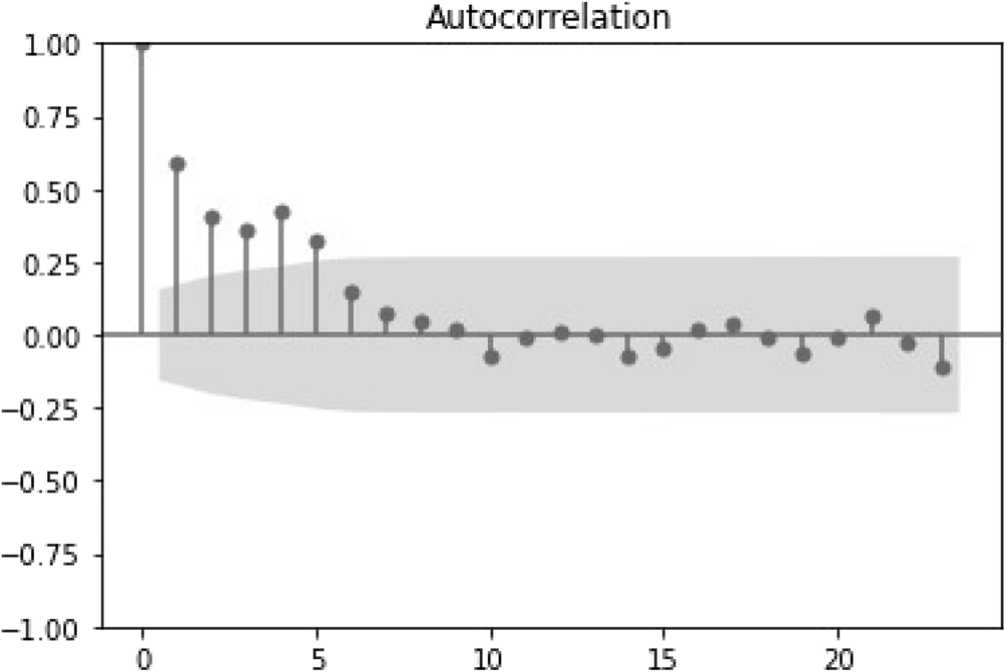
Autocorrelation plot for the time series of the weekly living donors.

For the first 12 lags, correlation is linearly decaying, confirming a slow drop in correlation for observations that are far apart. However, it is noted that the decay is much faster within the first 3 lags, after which 95% of the temporal correlation is already lost (see Fig. 5). With regard to the weekly time series, the correlation drops abruptly with only 7 lags falling outside the 95% confidence interval. As expected, correlation behaviours are different when zooming from years (and cumulative data) to weeks (and point-wise defined data). Specifically, the yearly-based correlation is much smoother, while the weekly-based empirical correlation exhibits many irregularities, due typically to the higher frequency of the observations.

Differences are observed in the living donation auto-correlation plots (Figs. 7, 8). Specifically, Fig. 7 depicts the statistical significance with a shorter lag when compared to the deceased annual donors in Fig. 5. However, the auto-correlation plot of the high frequency data (Fig. 8, weekly data) reflects some randomness in the time series and lack of correlation among its elements. This was not investigated further in the current study.

### Auto-regressive integrated moving average (ARIMA) model

ARIMA modeling has proved successful in a number of applications from several branches of applied sciences^15^. ARIMA generality and flexibility allow for a deep understanding of the structural components of a time series. Further, ARIMA models allow for the implementation of the prediction of a time series at unsampled (future) temporal instants, and in turn, this allows for the comparison against available data and clearer interpretations of the effect of the COVID-19 pandemic on organ donations.

For the yearly data, an ARIMA(*p*, *d*, *q*) with drift is used. Drift is incorporated in the ARIMA model to optimize a loss function as discussed below. This is a reasonable and sufficiently general model to tackle this kind of data set. Hence, we observe that the time series we are trying to fit is somewhat smoothened. Conversely, for the weekly data (observed at a much higher frequency), an ARIMA(*p*, *d*, *q*)(*P*, *D*, *Q*) has been used and its performance compared against an ARIMA(*p*, *d*, *q*) with drift.

#### Deceased and living donors annual data

For the annual time series, the following strategy was adopted. Both deceased and living donors were modeled according to an ARIMA time series, using the time frame 1988–2019. Then, the fittings from those time series were used to provide predictions for the years 2020, 2021, and 2022.

For fitting purposes, the Hyndman–Khandakar algorithm^16^ was used minimizing the Akaike Information Criterion (AIC) for the estimates that were in turn obtained through maximum likelihood estimation (MLE).

Based on the above criterion, an ARIMA(1,1,0) and ARIMA(0,1,1), both with drift, were obtained for the deceased and living organ donors, respectively.

The Akaike Information Criterion (AIC), an estimator of the prediction error and hence of the quality of the statistical model^19^, along with the root mean square error (RMSE), one of the standard measures of the error of a model in predicting quantitative data^20^, and mean absolute percentage error (MAPE), the mean or average of the absolute percentage errors of forecasts, are reported.

The AIC and RMSE might not be very reflective alone but are better understood when compared to the reported values of other models. MAPE, however, can be utilized to calculate the accuracy, as the complement of the relative error, where: Accuracy = 100 − MAPE^21^. Thus, the accuracy for ARIMA(1,1,0) with drift is 97.17% and of ARIMA(0,1,1) with drift is 96.14% with MAPE 2.832 and 3.860, respectively.

**Figure 9:**
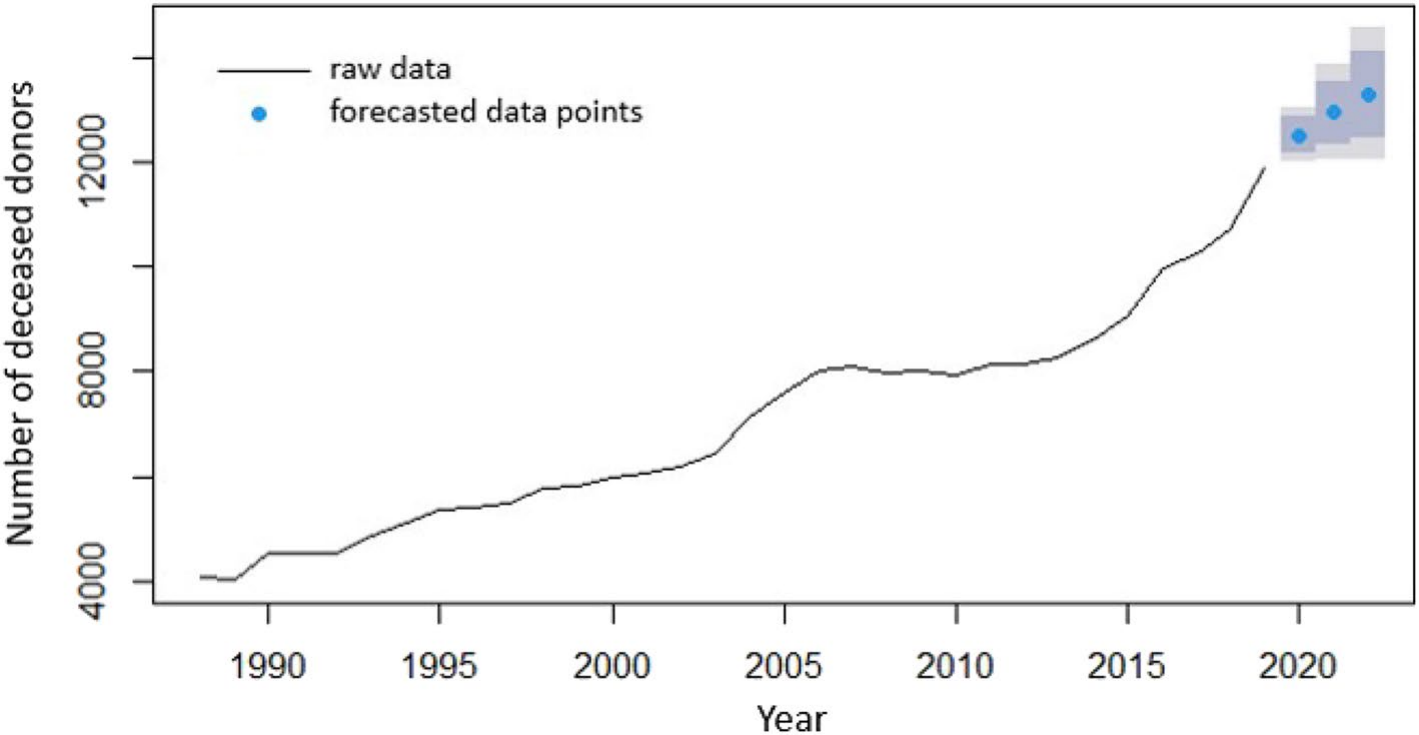
Raw data for deceased organ donors and forecasted data (blue dots) depicting predictions by the ARIMA model for years 2020 through 2022. Also shown are the 80% confidence interval (dark grey) and the 95% confidence interval (light grey).

**Figure 10:**
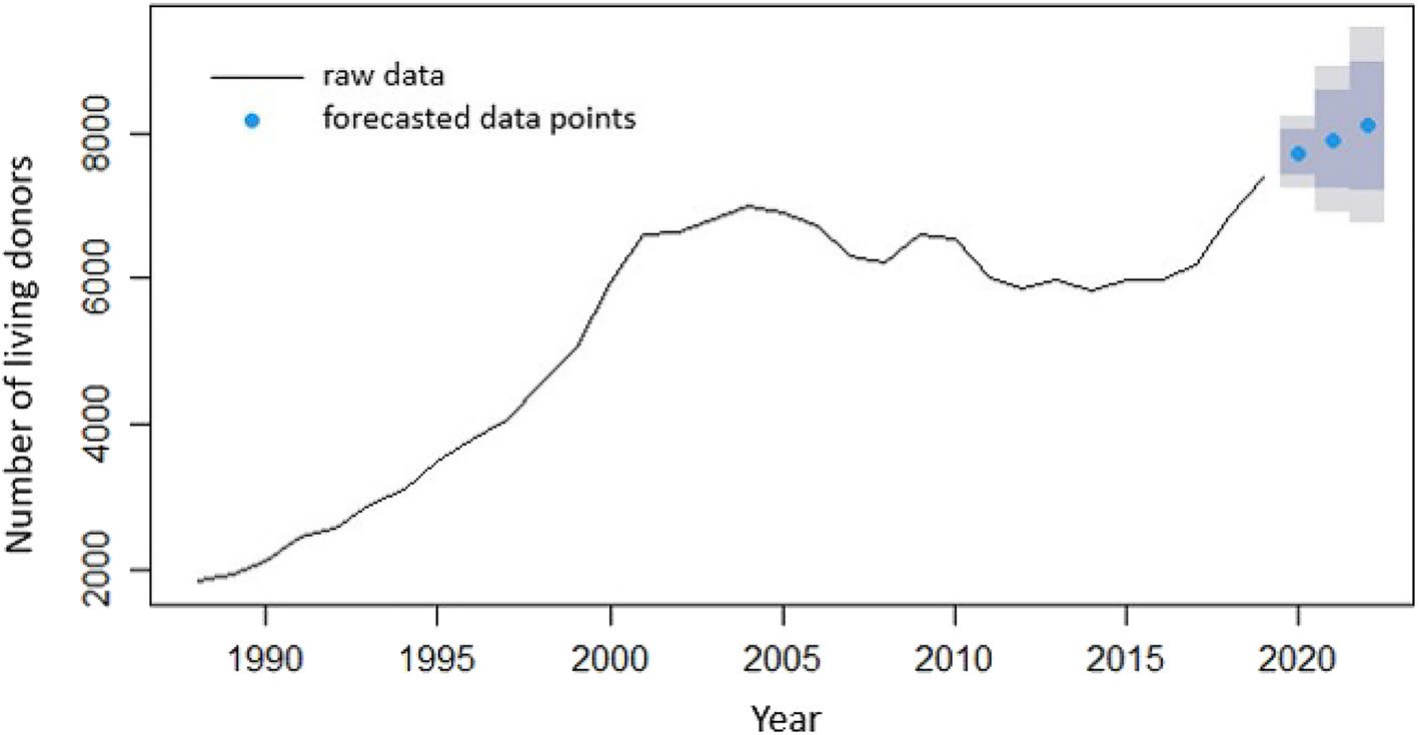
Raw data (pointwise data points connected by a line to show the change in the number of donors) for living organ donors and forecasted data (blue dots) depicting predictions by the ARIMA model for years 2020 through 2022. Also shown are the 80% confidence interval (dark grey) and the 95% confidence interval (light grey).

Figures 9 and 10 show the forecasted data for the years 2020 through 2022 in concert with the 95% confidence interval for the models fitted to the deceased and living organ donors, respectively.

The ratio of the observed to the expected data is also calculated as in Suarez-Pierre et al.^12^ and presented in Table 3.

#### Deceased and living donors weekly data

The analysis of the two weekly time series (living and deceased donors) was performed according to two alternative modeling strategies, denoted M1 and M2, respectively. The first model, M1, is developed on the basis of the same optimization criteria (AIC and MLE) explored for the case of yearly date. The second, M2, is based on ARIMA modeling of the remainders that are obtained by detrending and deseasonalizing the raw data.

For both strategies, the temporal horizon *week first of 2012* until *week 11th in 2020* is used as a training set. The validation set is instead the period *week 12th in 2020* to *week 16th in 2020* (4 weeks). We note that during those four weeks, the number of donors decreased significantly. The pandemic might be a cause of this.

**Figure 11:**
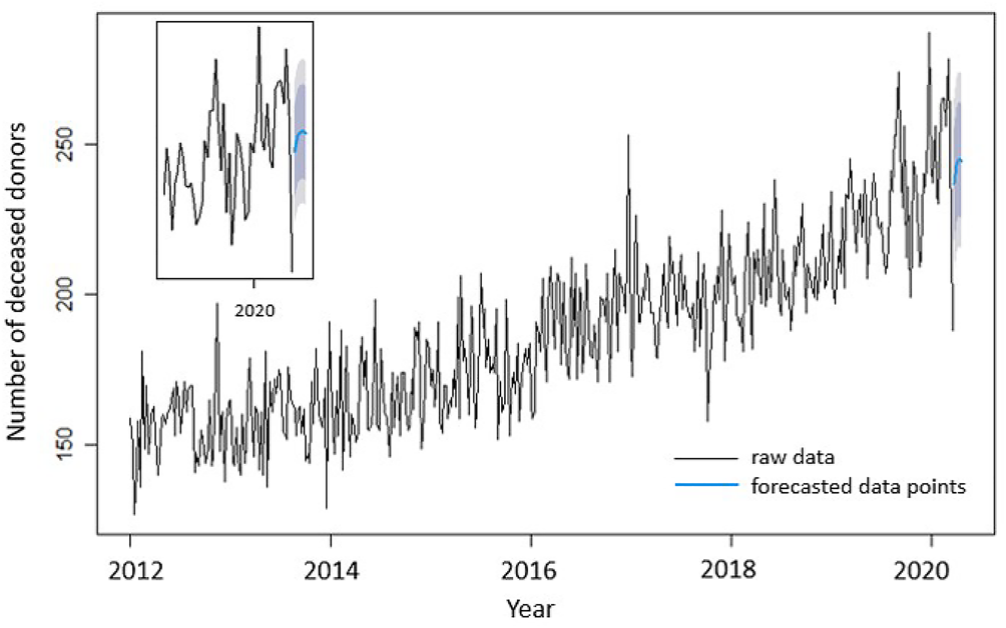
Raw data for the deceased organ donors along with blue line depicting the predictions by M1 ARIMA(0,1,2)(1,0,0) with drift. Also shown are the 80% confidence interval (dark grey) and the 95% confidence interval (light grey). The figure inset on the top left corner is a zoomed in view of the forecasted points and the confidence intervals.

Figure 11 depicts the raw data for the deceased organ donors along with the predictions by M1 ARIMA(0,1,2)(1,0,0) with drift. On the same plot, an inset with a zoomed-in view of the forecasted points and the confidence intervals is shown. The forecasted data points lie within the confidence intervals and predict an increase in the number of donors that peaks and then slightly decreases with time.

**Figure 12:**
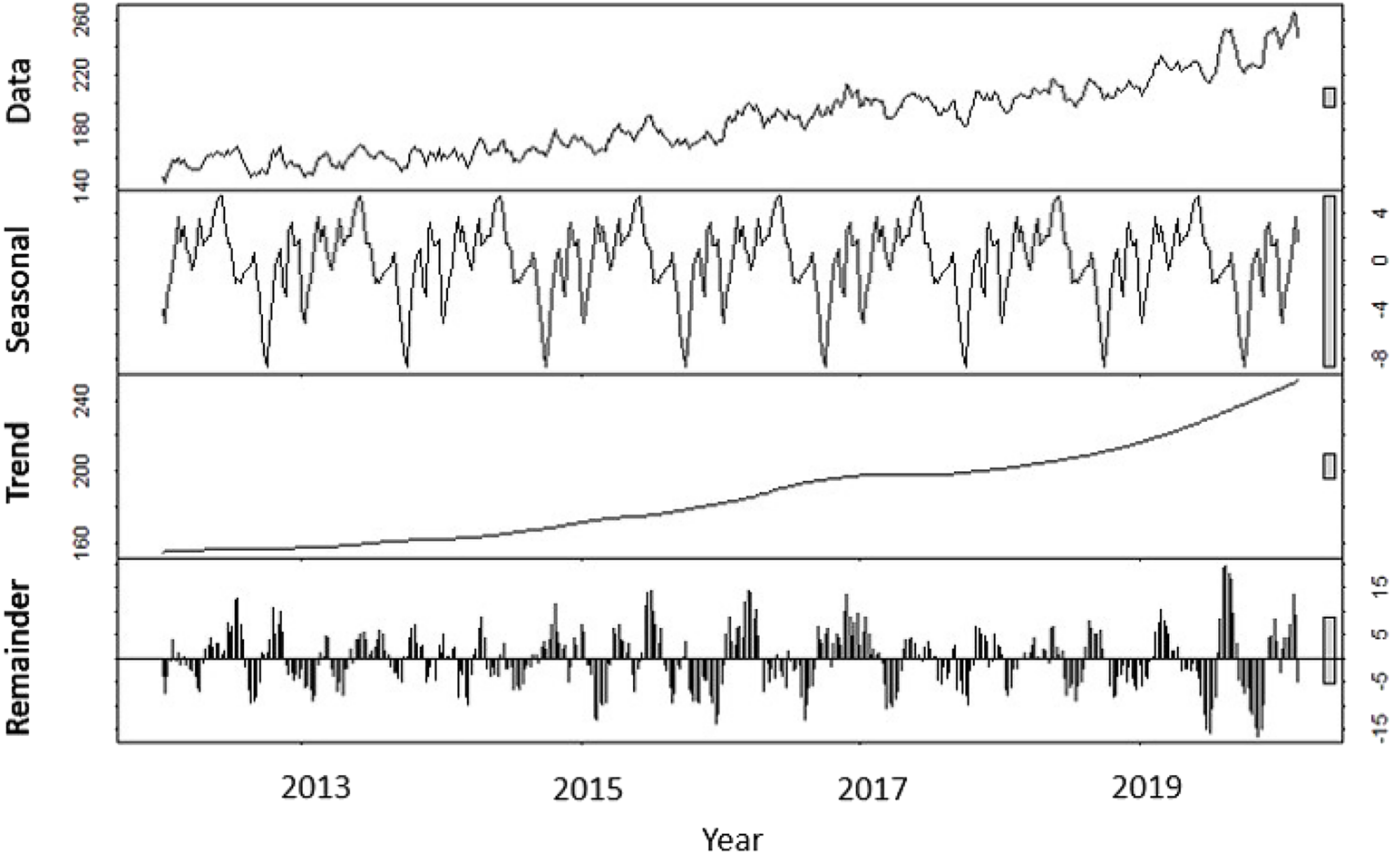
Raw data, seasonal component, trend, and the remainder when the raw data of deceased organ donors is adjusted by removing the seasonal component.

**Figure 13:**
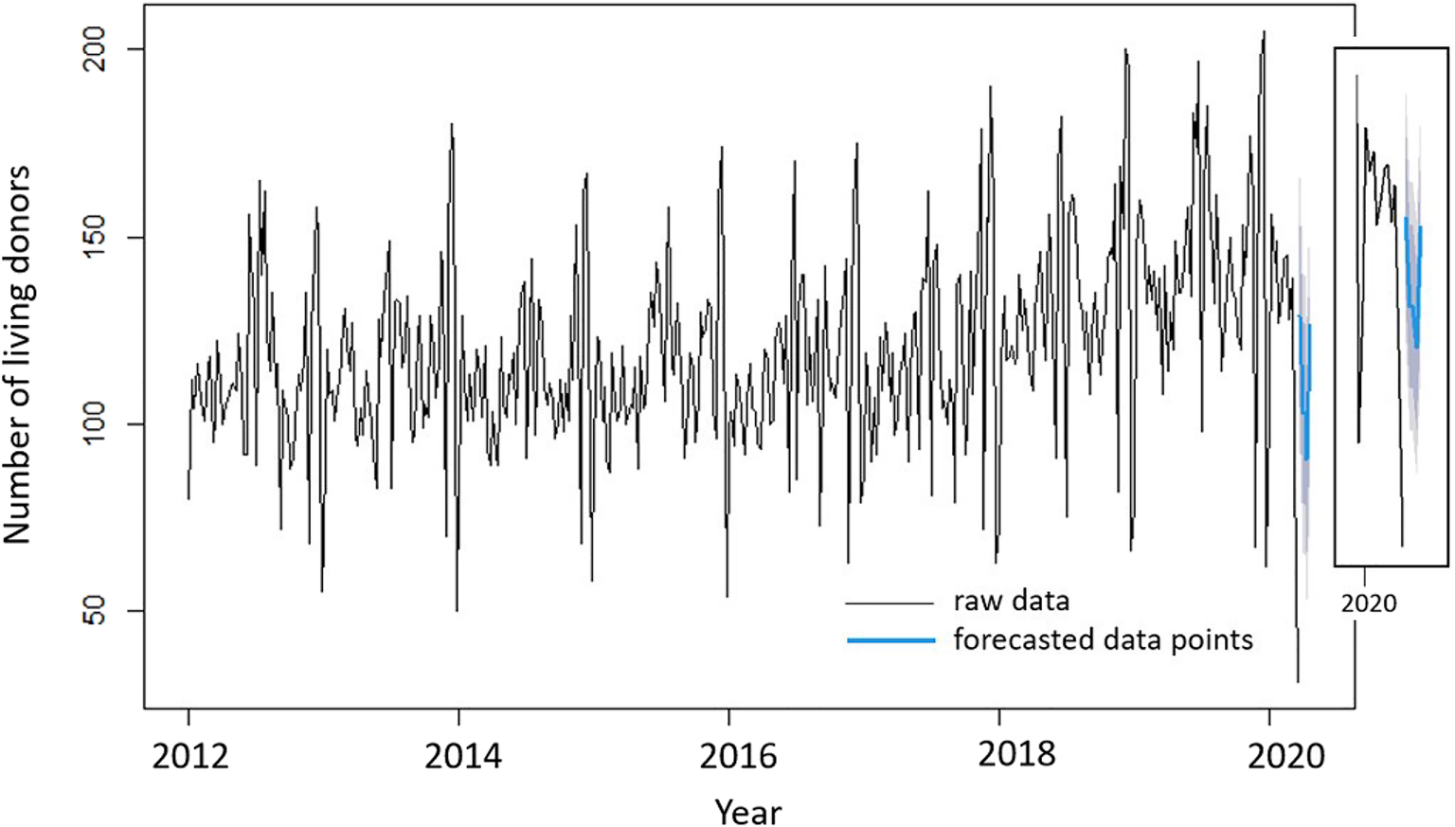
Raw data for the living organ donors along with blue line depicting the predictions by M1 ARIMA(6,1,0)(2,1,0) with drift. Also shown are the 80% confidence interval (dark grey) and the 95% confidence interval (light grey). The top right figure inset highlights the forecasted points and the confidence interval.

**Figure 14:**
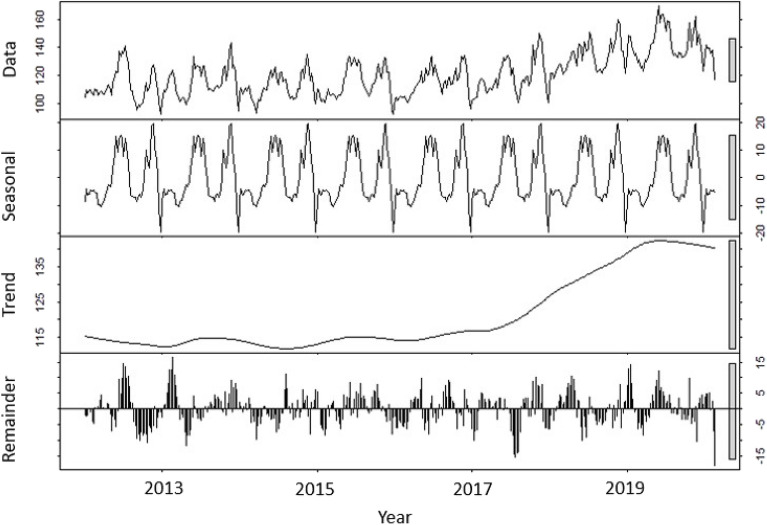
Raw data, seasonal component, trend, and the remainder when the raw data of living organ donors is adjusted by removing the seasonal component.

In Fig. 12, the effect of removing the seasonal component is highlighted.

Table 1 shows that the modeling approach M2, for this specific data set, is preferable in terms of both statistical accuracy and prediction.

**Table 1:** Comparison of the two different ARIMA models for deceased donors: Model M1 with seasonality (ARIMA(0,1,2)(1,0,0) with drift) and Model M2 with seasonally adjusted data constructed by removing the seasonal component (ARIMA(5,1,0) with drift).

	M1	M2
AIC	3505.99	2174.27
RMSE	14.45022	3.099693
MAPE	6.196596	1.332338

Figure 13 depicts the raw data for the living organ donors along with the predictions by M1 ARIMA(6,1,0)(2,1,0) with drift. On the same plot, an inset with a zoomed-in view of the forecasted points and the confidence intervals is shown. The model succeeds in predicting a drop in the number of living organ donors during the period when a surge in the number of cases due to the COVID-19 pandemic was detected. The forecasted data points all lie within the confidence intervals.

In Fig. 14, the effect of removing the seasonal component from the raw data of living organ donors is observed.

The results reported in Table 2, for the living donors, present consistent conclusions with those reported in Table 1, for the deceased donors. M2 uniformly outperforms M1 with respect to the three indicators as described above, thus, modeling strategy M2 provides a better assessment of statistical estimation and prediction.

**Table 2:** Comparison of the two different ARIMA models for living donors: Model 1 with seasonality (ARIMA(6,1,0)(2,1,0)) with drift) and Model 2 with seasonally adjusted data constructed by removing the seasonal component (ARIMA(0,1,5) with drift).

	M1	M2
AIC	3290.84	2149.67
RMSE	17.49363	3.003001
MAPE	11.20903	1.910031

Table 3 depicts the ratio between observations, denoted O (refer to the description of the training set), and predictions, denoted M$*$, with $*$ being either 1 or 2 in reference to the two modeling strategies adopted in this paper. The results are presented for both deceased and living donors. All obtained ratios lie within their prescribed 95$\%$ confidence bands. The most significant drop is appreciated for the living donors at week 13.

**Table 3:** Comparison of the two different ARIMA models for living donors: Model 1 with seasonality and Model 2 with seasonally adjusted data constructed by removing the seasonal component.

Week number	Deceased donors		Living donors	
	O/M1	O/M2	O/M1	O/M2
12	0.7932	0.7673	0.2403	0.2541
13	0.642	0.6393	0.1456	0.1181
14	0.6844	0.6987	0.1942	0.1613
15	0.6735	0.6875	0.17778	0.125
16	0.8197	0.8097	0.1587	0.146

## Discussion

The number of deceased organ donors in the USA does not appear to be negatively impacted by the COVID-19 pandemic (Fig. 1). This might be due to the fact that yearly data are a smoother version of the weekly data, and this fact was persistent through the analysis for both the deceased and living organ donors.

In Fig. 2, the drop in the number of USA deceased donors observed during weeks 65–67 is specifically due to the COVID surge that occurred.

Despite the fact that the pandemic continues to date, the data from the USA deceased organ donors is exemplary, when compared to the worldwide trend of organ donations, as it attained a peak in the weekly deceased donors of 309 for the first time at cumulative week number 122 (or week 18 in 2021 which spans from May 3 through May 9, 2021) as seen in Fig. 2. The data presented in this paper reflects the adaptability of the system and the commitment to saving lives. Another reason for the reduced impact of the COVID-19 pandemic on transplantation could be the opiate epidemic in North America. In five Canadian provinces from 2014 to 2017, there was a 35% increase (554–747) in total deceased organ donors but a 294% increase (31–122) in organ donors dying of an overdose and most commonly involving opiates^22^. At least 30 states in the USA have reported increases in opioid fatalities since the start of the pandemic^23^.

On the other hand, and as noted in Fig. 4, the decrease in the number of living donors starting from cumulative week number 64 till 71 (or weeks 12–19 in 2020 spanning from March 16 till May 10, 2020) is reflective of the strain imposed on the medical system during the pandemic where most (if not all) of the resources were allocated for COVID-19 patients. Another reason for this decrease in the number of living donors might have been the suspension of some living donation programs to avoid putting living donors at risk of contracting COVID-19 while hospitalized for an elective surgical procedure, and enhanced screening assessments for living donor candidates (i.e., requiring donors to be fully COVID-19 vaccinated). Some hospitals declined the opportunities for organ donation because of staffing shortages and a decrease in blood products were affected by COVID-19^24^.

In accordance with USA CDC guidance, transplantation is considered tier 3b (“do not postpone”)^25^. The American Society of Transplantation recommends that kidneys from donors with an intermediate risk of COVID-19 infection can be considered with caution^9^. They suggested that it is safe to use organs from donors who are low-risk or COVID-19 negative, as well as those who have recovered from COVID-19 disease more than 28 days before donation^9^. Others have witnessed the safety of transplantation of kidneys from COVID-19 positive donors^26^. However, the decision to proceed with transplantation was determined by each transplant center. In many cases, living liver donation was deferred. Instead, deceased donor liver transplantation was pursued^24^. This study^24^ summarizes as well some of the negative impacts of the ongoing pandemic on organ recovery and liver transplantation. Several recommendations and different plans to mitigate these negative effects are suggested and might be found efficient even after the infections with the coronavirus disease 2019 have declined.

Another worldwide organ transplantation study (kidney, liver, lung, and heart transplants) conducted in 22 countries, including the USA, shows that there has been an overall decrease of 15.92$\%$ in organ transplant. Specifically, in the USA the decrease amounts for 4.13$\%$ from the date of the 100$^{th}$ reported cumulative COVID-19 case to Dec 31, 2020 (or the end of follow-up, whichever is earlier) to the same period of time in 2019^27^.

The American Society of Transplant Surgeons recommends suspending all living donations unless necessary^9^. An International survey^28^ on living kidney donation and transplant practices during the COVID-19 pandemic shows the evaluation of donor candidates was significantly reduced during the COVID-19 pandemic, with 59% of programs pausing living donor evaluation. Additionally, in a study conducted in the United Kingdom^29^, Sharma et al. say that the reduction in transplant activity results in a total of 720–1438 patients continuing to rely on dialysis instead of otherwise having an organ transplant. Centers in the United States that continued living donor evaluations generally used video-based assessment (68%) or telephone-based contact (42%). However, 40% of centers still used in-clinic assessment (up to 83% among the centers located outside of North America)^28^.

With the ongoing COVID-19 pandemic, some countries such as Italy, are considering organs from donors with active SARS-CoV-2 infection, as well as donors with asymptomatic COVID-19 infection who died from causes unrelated to COVID-19^30^.

## Study limitations

Further analysis could explore whether calibration of the two time series with different resolutions might provide additional insight. This is beyond the scope of the present work.

## Conclusions

In the USA, organ transplantation has been affected by the Covid-19 pandemic. Specifically, living organ donation has experienced a bigger negative impact than deceased donations; however, this mathematical study shows the adaptability of the USA healthcare system to pivot to address the COVID-19 pandemic while still managing transplantation and thus it could potentially help other countries facing pandemic impacts to their solid organ transplant programs. A county’s pandemic response will always be shaped by numerous factors that are always country specific such as regulations, healthcare economics, and cultural values and practice.

## Data Availability

The data analyzed in this study is available from the OPTN Organizational Metrics Dashboard^13^. This dashboard is a series of interactive dashboards providing high-level information regarding trends in transplantation and donation. The dashboard allows users to investigate current and historic data across all organ groups and select specific OPTN regions, donor types, timeframes, etc. The dashboard is accessible via https://optn.transplant.hrsa.gov/ under the Data section. For data feedback or questions, please contact OPTN at DataProducts@unos.org. For additional inquires on the specific data used for this study, please contact the corresponding author.
